# Cross-dataset late fusion of Camera–LiDAR and radar models for object detection

**DOI:** 10.1038/s41598-025-32588-5

**Published:** 2026-01-07

**Authors:** Assem Ali, Mohamed M. Tawfik, Mahmoud M. Saafan

**Affiliations:** 1 Mechatronics Engineering Program, Faculty of Engineering, Mansoura University, Mansoura, Egypt; 2 Mechanical Power Engineering Department, Faculty of Engineering, Mansoura University, Mansoura, Egypt; 3 Computers and Control Systems Engineering Department, Faculty of Engineering, Mansoura University, Mansoura, Egypt; 4 Faculty of Engineering, Mansoura National University, Mansoura, Egypt

**Keywords:** Engineering, Mathematics and computing

## Abstract

This paper presents a modular late-fusion framework that integrates Camera, LiDAR, and Radar modalities for object classification in autonomous driving. Rather than relying on complex end-to-end fusion architectures, we train two lightweight yet complementary neural networks independently: a CNN for Camera + LiDAR using KITTI, and a GRU-based radar classifier trained on RadarScenes. A unified 5-class label space is constructed to align the heterogeneous datasets, and we verify its validity through class-distribution analysis. The fusion rule is formally defined using a confidence-weighted decision mechanism. To ensure statistical rigor, we conduct 3-fold cross-validation with three random seeds, reporting mean and standard deviation of mAP and per-class AP. Results show that the Camera + LiDAR model achieves a strong average mAP of 95.34%, while Radar achieves 33.89%, reflecting its robustness but lower granularity. Using the proposed late-fusion rule, performance increases to 94.97% mAP versus KITTI ground truth and 33.74% versus RadarScenes. Cross-validated per-class trends confirm complementary sensing: Camera + LiDAR excels at Cars, Bicycles, and Pedestrians, while Radar contributes stability under adverse conditions. The paper also provides a complexity and latency analysis, discusses dataset limitations, clarifies temporal handling for radar, and includes updated literature up to 2025. Findings show that lightweight late fusion can achieve high reliability while remaining computationally efficient, making it suitable for real-time embedded autonomous driving systems.

## Introduction

Autonomous driving systems rely heavily on accurate and robust perception of the surrounding environment. Cameras provide high-resolution semantic information but are sensitive to illumination changes, shadows, and adverse weather conditions such as fog or heavy rain. LiDAR offers precise 3D geometric structure, yet its performance may degrade on reflective surfaces or at long range. Radar sensors, in contrast, maintain reliability in poor visibility but have low spatial resolution and higher measurement noise. Because each sensor exhibits complementary strengths and inherent weaknesses, recent studies have emphasized that multi-sensor fusion is essential for achieving reliable perception in real-world autonomous driving^1–3^. Fusion enables systems to combine semantic detail, geometric accuracy, and environmental robustness, ultimately leading to more dependable object detection and tracking performance.

Sensor fusion strategies are commonly classified into early, middle, and late fusion, each with distinct advantages and limitations. Early fusion combines raw sensor data (e.g., pixel values and point clouds) before neural processing. This approach can exploit low-level correlations between modalities, but it requires strict temporal and spatial calibration and is computationally expensive—challenges highlighted in works such as BEVFusion^4^ and DeepFusion^5^. Middle fusion merges intermediate feature representations extracted from each modality. Methods such as TransFuser^6^ and UniFusion^7^ demonstrate strong performance by learning cross-modal attention and geometric alignment, but they depend on tightly coupled architectures and require complete multi-modal data during training. Late fusion, the approach adopted in this study, combines final predictions or high-level outputs from independently trained models. Recent research shows that late fusion offers flexibility, modularity, and robustness to missing or degraded sensors, making it practical for heterogeneous datasets or systems with variable sensing conditions^2,8^. This strategy is particularly useful when datasets do not share identical sensor modalities—as in the case of KITTI (Camera + LiDAR) and RadarScenes (Radar)—because independent models can be trained on each dataset and fused at the decision level.

**Fig. 1:**
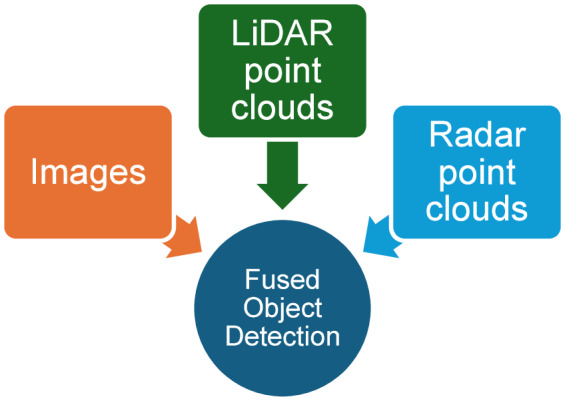
Graphical summarization of the purpose of the study.

**Fig. 2:**
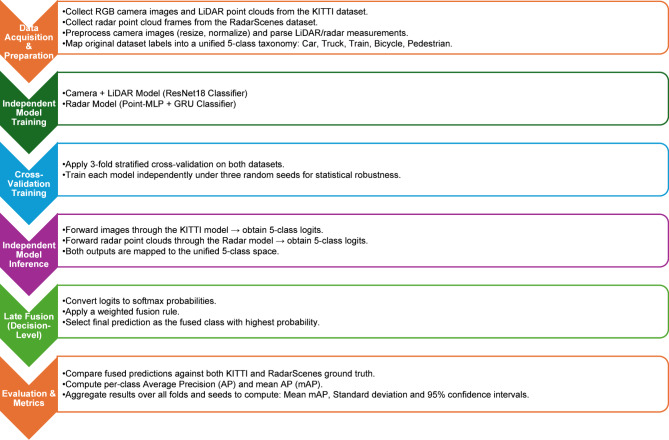
Graphical summarization of working mechanism of the framework.

In this work, we build upon these insights and propose a late fusion framework to improve object detection by combining Camera, LiDAR, and Radar sensors. The motivation stems from recent findings showing that combining visual and geometric cues with radar-based motion and robustness features significantly enhances performance, especially in adverse conditions or ambiguous scenes^3,5,8^. Figure 1 summarizes the overall purpose of our study, and Fig. 2 illustrates the working mechanism of the proposed framework. By integrating these complementary modalities through a lightweight and modular design, our approach aims to improve detection reliability while remaining computationally efficient.

The main contributions of the current study are:

- Develop a CNN model designed to process RGB camera images and LiDAR features for object detection.
- Develop an RNN model designed to process variable-length radar point cloud data for classification.
- Use two different datasets for models training; KITTI for camera and LiDAR, RadarScenes for radar.
- Develop a late fusion framework that utilizes predictions from the camera + LiDAR, and radar models.
- Report experimental results compared to other methods using mAP performance metric.

The rest of the paper is divided into these sections. Section II discusses the related work in using different sensors for the goal of object detection. Datasets used and our methodology are described in Section III. In Section IV, the experimental setup and results obtained out of the experiments are reported. Results are further described and discussed in Section V. Section VI presents the conclusions and future work.

## Related work

Research in multi-sensor fusion for autonomous driving has accelerated rapidly in recent years, driven by the need for robust perception under diverse environmental conditions. Modern fusion pipelines integrate complementary sensing modalities—typically cameras, LiDAR, and radar—to improve detection reliability and spatial reasoning.

A massive effort has been made in LiDAR-based object detection, efficiently extracting 3D features from point clouds. PointPillars^9^ introduces a novel approach to 3D object detection using LiDAR point clouds by proposing a pillar-based encoding scheme. The method leverages a simplified encoder that enables end-to-end learning while achieving competitive accuracy on the KITTI benchmark achieving 59.20% mAP.

SECOND^10^ presents an efficient 3D object detection framework that builds upon voxel-based representations of LiDAR point clouds. It demonstrates strong performance on the KITTI benchmark, balancing accuracy and efficiency of 76.48%. CenterPoint^11^ is a unified framework for 3D object detection and tracking that localizes object centers in the bird’s-eye view (BEV) space. Instead of detecting bounding boxes directly, CenterPoint reformulates detection as a keypoint estimation problem—predicting the center of each object followed by regressing its attributes (size, orientation, velocity). It utilized Waymo dataset and achieved a 59.30% mAP.

VoxelNet^12^ was one of the first frameworks to introduce a fully end-to-end deep learning pipeline for 3D object detection directly from raw LiDAR point clouds. It divides the 3D space into voxels and uses a Voxel Feature Encoding (VFE) layer to learn point-wise and voxel-wise features jointly.

A major trend in 3D object detection is the use of Bird’s-Eye View (BEV) fusion, where heterogeneous sensor features are projected into a unified spatial representation.

BEVFusion^4^ introduced a unified BEV encoder that jointly fuses LiDAR point clouds and multi-view camera images for multi-task perception. This architecture preserves geometric accuracy from LiDAR while leveraging camera semantics, producing strong performance with reduced inference cost.

UniFusion^7^ further extended BEV-based fusion through a transformer-based architecture that fuses both spatial and temporal cues across multiple camera views. By unifying feature representations before detection, BEV-transformer methods achieve high accuracy but require synchronized multi-modal data and substantial computational resources.

The main advantage of these BEV fusion approaches is their ability to encode multi-modal information within a shared coordinate system, improving localization and depth perception. However, they often suffer from high memory costs, reliance on dense LiDAR data, and reduced robustness when one modality becomes degraded or unavailable.

For camera-based object detection, convolutional neural networks (CNNs) like Faster R-CNN^13^ achieves state-of-the-art performance on benchmarks such as COCO (42.7% mAP), and set a new standard for two-stage object detectors. While originally developed for 2D images, its core ideas influenced many 3D detection architectures in autonomous driving, especially those incorporating camera data.

YOLO^14^ presents enhancements to the YOLO (You Only Look Once) series of single-stage object detectors, focusing on improving accuracy while maintaining real-time speed. Although it sacrifices some accuracy (28.2%) compared to two-stage detectors like Faster R-CNN, YOLOv3 achieves high inference speed, making it well-suited for time-sensitive applications such as autonomous driving. Those have been widely adopted due to their effectiveness in real-time tasks.

Radar-based methods have received less attention historically but are gaining traction for their robustness in low-visibility conditions. Recent research^8^ provides a comprehensive overview of how radar sensors are being integrated into autonomous driving systems using deep learning techniques. The review concludes that while radar has strong potential due to its robustness in adverse weather and low cost, significant research is needed to fully exploit its capabilities using deep learning which emphasizes the potential of radar in autonomous driving, especially in adverse weather.

Radar’s robustness in fog, rain, darkness, and glare has motivated an increasing number of radar-vision fusion approaches. A recent survey on radar and vision methods^15^ provides a detailed taxonomy of deep-learning-based fusion strategies, demonstrating radar’s growing importance in robust perception.

MVFusion^16^ proposed semantic-aligned camera–radar fusion using a cross-attention transformer that aligns radar and camera features at the semantic level. This method showed that radar can effectively complement cameras by providing range, velocity, and occlusion-resilient cues. Despite these benefits, radar’s sparse and noisy structure creates challenges in accurately aligning features with image data.

Camera–radar fusion generally excels in adverse weather and long-range detection but remains limited by low spatial resolution, calibration sensitivity, and sparsity in radar point clouds.

Several approaches to sensor fusion in autonomous driving have been proposed. Fusion techniques vary widely; some methods use early fusion to combine raw sensor data before feeding it into a single model, while others employ late fusion, combining individual sensor outputs at the decision stage.

Researches have been conducted on fusion methods like RVF-Net^17^ which proposes a novel early fusion strategy that integrates radar and LiDAR data at the voxel level to enhance 3D object detection. The paper demonstrates that radar-augmented voxel features improve performance over LiDAR-only baselines on datasets such as nuScenes by achieving 54.86% mAP. RadarNet^18^ presents a deep learning framework that leverages automotive radar data to detect and classify dynamic objects in complex driving environments. It demonstrates competitive performance compared to LiDAR-based methods of 60.40% mAP, especially for detecting small or occluded objects.

On the other hand, Bi-LRFusion^19^ introduces a novel bi-directional fusion architecture that enables mutual enhancement between LiDAR and radar modalities for dynamic object detection in autonomous driving. The framework is particularly effective in identifying moving objects and maintaining performance under challenging conditions such as fog or rain.

More recent research has explored hybrid architectures that combine LiDAR, radar, and sometimes camera data.

For example, a LiDAR + 4D radar fusion pipeline proposed in^20^ introduces an adaptive gating mechanism that modulates radar contributions depending on scene conditions. This increases robustness when either modality becomes unreliable.

Another modern fusion approach, DifFUSER^21^, leverages diffusion models to fuse multi-modal features and generate robust BEV representations. By using generative refinement, the model can recover missing or corrupted modality information, improving perception stability under degradation.

Hybrid fusion methods tend to achieve the highest robustness, but their complexity, training cost, and requirement for synchronous multi-sensor datasets can limit practical deployment.

Table 1 shows a summary of the mentioned methods used in object detection. Our approach builds on late fusion techniques, enabling independent sensor models and allowing flexible fusion strategies.

**Table 1:** Summary of methods used in object detection.

Method	Modality	Dataset	mAP	Pros.	Cons.
PointPillars^9^	LiDAR	KITTI	59.20%	- Fast and efficient due to pillar encoding- Real-time inference - Good for 3D bbox	- Loses some fine-grained vertical info - Performance can degrade in sparse scenes
SECOND^10^	LiDAR	KITTI	76.48%	- Sparse 3D convolution reduces computation - Strong 3D detection accuracy	- Still computationally heavier than PointPillars - Complex to tune
CenterPoint^11^	LiDAR	Waymo	59.30%	- State-of-the-art accuracy - Detects objects as keypoints (center), boosting precision	- More resource-intensive - May need post-processing refinements
VoxelNet^12^	LiDAR	KITTI	N/A	- End-to-end 3D detection - Preserves 3D structure well	- Very high computational cost - Not real-time
Faster R-CNN^13^	Camera	COCO trainval	42.7%	- High accuracy in 2D detection - Works well on high-res images	- Slow inference - Poor depth understanding, lacks 3D info
YOLO^14^	Camera	COCO trainval	28.2%	- Very fast inference - Suitable for real-time detection - Lightweight	- Less accurate than R-CNN-based models - Limited small object detection
RadarNet^18^	LiDAR + Radar	nuScenes	60.40%	- Robust to adverse weather/light - Handles occlusion well	- Low spatial resolution - Needs strong post-processing to reduce false positives
Bi-LRFusion^19^	LiDAR + Radar	nuScenes	62.00%	- Combines dense + robust perception - Improves detection of dynamic objects	- Complex architecture - Sensor calibration issues may arise
RVF-Net^17^	Camera + LiDAR + Radar	nuScenes	54.86%	- Enhances camera with motion cues - Good for adverse conditions	- Radar-camera alignment critical - Performance is heavily dependent on dataset
BEVFusion^4^	Camera + LiDAR	nuScenes	69.1%	- Unified BEV representation for all modalities. - Strong performance across detection and segmentation. - Efficient and scalable for multi-task perception.	- Requires precise calibration. - Heavy GPU requirements. - Performance relies on LiDAR quality.
UniFusion^7^	Camera + LiDAR + Radar	nuScenes, Waymo	NA	- Unified transformer backbone across modalities. - Handles missing or corrupted sensor data robustly. - Strong generalization across datasets.	- Full transformer stack is computationally expensive. - Not optimized for real-time deployment.
MVFusion^16^	Camera + LiDAR + Radar	nuScenes	61.7%	- Multi-view cross-attention improves modal interaction. - Handles occlusion and complex scenes better than dual-modal fusion. - Combines 2D, BEV, and 3D cues.	- High computational overhead due to multi-view processing. - Requires careful synchronization of three modalities.
LiDAR + 4D Radar Distillation^20^	LiDAR + 4D Radar	Waymo, RoboSense	68.4%	- Uses radar motion cues to enhance point-cloud quality. - Distillation reduces ambiguity in 4D radar signals. - Robust under rain/fog conditions.	− 4D radar is still expensive and uncommon. - Distillation requires paired LiDAR + radar data.
DifFUSER^21^	Camera + LiDAR + Radar	nuScenes	66.3%	- Diffusion-based fusion improves robustness to missing data. - Strong performance under sensor dropout tests. - Flexible architecture for modality-agnostic fusion.	- Diffusion models add latency (slow inference). - Training is computationally intensive.

## Methodology

### Data acquisition and description

The current study utilizes two types of datasets. The first type is KITTI dataset^22^ which is a benchmark widely used in autonomous driving research. It contains synchronized data from cameras and LiDAR sensors, with detailed annotations for object detection. We utilize the RGB images and point cloud data from the dataset to train the model. On the other hand, the second type is RadarScenes dataset^23^ which focuses exclusively on radar data and provides comprehensive annotations for longer-range object detection. This dataset is particularly useful for detecting objects in poor visibility, a scenario where both camera and LiDAR often struggle. We use this dataset to train our radar-specific model.

### Data pre-processing

#### KITTI pre-processing

The KITTI Camera–LiDAR samples are pre-processed to ensure consistent input formatting for the ResNet-based classifier. RGB images are first converted to PyTorch tensors and normalized using ImageNet statistics($\:\mu\:=[0.485,\:0.456,\:0.406]$, $\:\sigma\:=\:[0.229,\:0.224,\:0.225\:])$, which maintains compatibility with the pretrained ResNet-18 backbone. Images are resized to a unified spatial resolution of $\:256\:\times\:\:832$, ensuring consistent batch formation during training.

Annotation files are parsed to extract object categories, which are then mapped into the unified 5-class space used throughout the framework. Only the class labels are used in this study; LiDAR point clouds are not directly fused into the model but are retained within the dataset for future extensions.

All samples are partitioned using a stratified 3-fold cross-validation scheme (80% training, 20% testing within each fold), preserving class distribution across splits and reducing potential dataset bias.

#### RadarScenes pre-processing

RadarScenes data is stored in an HDF5 structure and consists of radar point clouds and associated motion information. Each radar sweep is processed as follows:

1. **Data Extraction**:

Radar point returns and odometry measurements (e.g., ego-vehicle velocity and yaw rate) are loaded and associated using timestamp alignment to ensure that each radar frame incorporates its correct motion context.

2. **Feature Construction**:

For each radar point, nine physical features are extracted:

- **Polar coordinates**: range^sc^, azimuth^sc^.
- **Signal properties**: radar cross-section (RCS).
- **Velocities**: $\:{v}_{r}$, $\:{v}_{r,\:comp}$ (Doppler-compensated radial velocity).
- **Cartesian positions**: $$\:{x}_{cc}$$, $$\:{y}_{cc}$$, $$\:{x}_{seq}$$, $$\:{y}_{seq}$$

These attributes are concatenated into a feature matrix of shape $\:({N}_{points},\:9)$, allowing variable-length radar frames.

3. **Label Mapping**:

Original RadarScenes categories (12 classes) are mapped into the unified 5-class label space. Dynamic objects not relevant to the five target classes are remapped to the nearest corresponding category or filtered if ambiguous.

4. **Dataset Assembly**:

Since each radar frame contains a variable number of returns, no spatial voxelization or temporal stacking is applied. Instead, each frame is treated as one radar “sample,” and its feature matrix and label are returned by a custom PyTorch Dataset.

This representation preserves the raw structure of radar sweeps, enabling the Radar GRU model to handle variable-length inputs using masked pooling and single-step temporal encoding.

### Models development

Two independent neural network models were developed to process the heterogeneous sensor modalities: a ResNet-based model for Camera + LiDAR inputs (KITTI) and a GRU-based model for radar point clouds (RadarScenes). The models are trained separately and fused later at the decision level.

#### KITTI ResNet classifier

The Camera + LiDAR branch uses a ResNet-18 backbone adapted for single-frame RGB image classification. The model processes normalized RGB images of size $\:3\:\times\:\:256\:\times\:\:832$3. LiDAR features are not explicitly fused inside the backbone; instead, the network focuses on extracting high-level semantic features from the camera stream, which aligns with the need for robust visual recognition.

The standard fully connected layer of ResNet-18 is replaced by a lightweight classification head consisting of:

- A 256-unit fully connected layer.
- ReLU activation.
- Dropout (*p* = 0.5).
- A final linear layer mapping to the unified 5-class label space.

An optional weight-freezing mechanism allows the backbone to remain fixed when pretrained weights are used. The architecture emphasizes feature reuse and low computational cost while maintaining competitive performance.

**Architectural Summary**.

- **Backbone**: ResNet-18 (pretrained on ImageNet).
- **Classifier head**: Linear(512→256) → ReLU → Dropout → Linear(256→5).
- **Output**: logits for 5 unified object classes.
- **Fusion option**: LiDAR placeholder argument retained for extensibility.

This design provides a practical middle ground between classical CNNs and heavier state-of-the-art fusion architectures.

#### Radar GRU classifier

Radar point clouds contain variable numbers of returns per frame and exhibit substantial noise. To handle this modality, a dedicated RadarScenes classifier based on per-point feature encoding and gated recurrent units (GRUs) was developed.

Each radar point contains nine features (range, azimuth, velocity, RCS, and Cartesian projections). These are processed through a per-point multilayer perceptron (MLP) to obtain a learned embedding for each radar return. A masked max-pooling operation aggregates variable-length point sets into a single frame-level feature vector.

To provide temporal modeling capability, even though each frame here is treated as a single time step, the pooled feature is passed through a GRU layer. This architectural choice allows easy extension to multi-frame radar sequences in future work.

The classification head consists of a 128-unit fully connected layer with ReLU and dropout, followed by a linear mapping to 5 output classes.

**Architectural Summary**.

- **Input**: variable-length radar point cloud $\:({N}_{points},\:9)$.
- **Per-point encoder**: MLP (9→64→64).
- **Aggregation**: masked max-pooling.
- **Temporal module**: GRU (input size 64, hidden size 128).
- **Classifier**: Linear(128→128) → ReLU → Dropout → Linear(128→5).
- **Output**: logits for 5 unified object classes.

This design allows the model to retain radar-specific structural and motion cues while remaining computationally efficient.

### Late fusion strategy

In the proposed framework, late fusion is performed at the decision level by combining the output probabilities of the Camera + LiDAR classifier and the Radar classifier. Both models independently produce logits over the same unified 5-class label space (Car, Truck, Train, Bicycle, Pedestrian) after applying the defined mapping functions for KITTI and RadarScenes labels. This ensures a consistent representation across heterogeneous sensing modalities.

During evaluation, paired batches from the KITTI and RadarScenes validation sets are processed in parallel. For each KITTI batch, RGB images and their corresponding LiDAR features are forwarded through the ResNet-based classifier, producing a logit vector $\:{z}_{kitti}\:\in\:{R}^{5}$. Simultaneously, the radar point-cloud batch is forwarded through the GRU-based radar network, yielding logits $\:{z}_{radar}\:\in\:{R}^{5}$.

Both logits are converted to class-probability distributions via the softmax operator:

$$\:{P}_{kitti}=softmax\left({z}_{kitti}\right),\:{P}_{radar}=softmax\left({z}_{radar}\right).$$

To combine the predictions, a probability-weighted late fusion rule is applied:

$$\:{P}_{fused}=0.6\:{P}_{kitti}+0.4\:{P}_{radar}$$.

This rule follows a fixed weighting scheme in which the Camera + LiDAR model is assigned higher influence (0.6) due to its superior baseline performance, while radar retains meaningful contribution (0.4) thanks to its robustness in adverse conditions. The result is a fused probability distribution whose class with maximum probability is selected as the final prediction:

$$\:{\widehat{y}}_{fused}=argmax{P}_{fused}$$.

Ground-truth labels are taken from the RadarScenes dataset for fusion evaluation, given that radar labels are temporally dense and consistent within each scene. KITTI and RadarScenes labels are converted to the unified 5-class space using the defined mapping dictionaries, ensuring alignment between modalities.

For each seed, all fused predictions and ground-truth labels are accumulated across the entire validation set. Per-class Average Precision (AP) is computed using one-vs-rest evaluation. The mean Average Precision (mAP) for each seed is then obtained as:

$$\:{mAP}_{seed}=\:\frac{1}{5}\sum\:_{c=0}^{4}{AP}_{c}$$.

To assess the statistical robustness of the fusion strategy, experiments are repeated independently for three random seeds (42, 2024, 7). The final reported score includes:

- the mean mAP across seeds,
- the standard deviation, and.
- the 95% confidence interval.

This multi-seed evaluation provides a rigorous quantification of performance variability and ensures that reported improvements are statistically meaningful rather than dependent on a single initialization. Table 2 describes Algorithm 1 of the pipeline in detail.

**Table 2:** Algorithm 1: late fusion and Multi-Seed evaluation Pipeline.

Step	Description
1. Initialization	• Select computation device (GPU/CPU). • Define seed set SEEDS = {42, 2024, 7}. • Define unified labeling functions map_kitti_labels(·) and map_radar_labels(·). • Set number of unified classes C = 5. • Define fusion weights wₖ = 0.6, w_r_ = 0.4.
2. Prepare Validation Data	• Create 80/20 KITTI split → validation loader with image & LiDAR pairs. • Create 80/20 RadarScenes split → validation loader with radar feature tensors. • Ensure both loaders iterate in parallel.
3. Per-Seed Loop	For each seed s ∈ SEEDS:
3.1 Load Models	• Load KITTI checkpoint kitti_model_checkpoint_seed{s}.pth. • Load Radar checkpoint radar_model_checkpoint_seed{s}.pth. • Instantiate models and set to eval().
3.2 Initialize Storage	• all_preds = [] • all_targets = []
3.3 Batch Processing	For each paired batch (KITTI_batch, Radar_batch):
3.3.1 KITTI Forward	• Extract images, lidars, class_ids. • Compute KITTI logits Lₖ = modelₖ(images, lidars). • Convert to probabilities Pₖ = softmax(Lₖ).
3.3.2 Radar Forward	• Extract radar_features, radar_labels. • Compute Radar logits L_r_ = model_r_(radar_features). • Convert to probabilities P_r_ = softmax(L_r_).
3.3.3 Batch Alignment	• Find common batch size B = min(Bₖ, B_r_). • Truncate KITTI and Radar outputs to size B.
3.3.4 Fusion Rule	Compute fused probability vector for each sample: P_fused_ = wₖ Pₖ + w_r_ P_r_ Append to all_preds.
3.3.5 Unified Target Mapping	• Map KITTI labels using map_kitti_labels. • Map Radar labels using map_radar_labels. • Use Radar’s unified labels as evaluation ground truth: unified_targets = T_r_. Append to all_targets.
4. After All Batches (Per Seed)	• Stack predictions and targets into arrays.
4.1 Compute Per-Class AP	For each class c = 0…4: • Create binary labels y_true = (targets = = c). • Scores y_score = fused_probs[:, c]. • If no positives → AP₍c₎ = NaN. • Else compute AP using sklearn’s average_precision_score.
4.2 Compute Seed mAP	• mAP_seed = nanmean(AP_vector) • Store mAP and AP results.
5. Multi-Seed Aggregation	After all seeds evaluated:
5.1 Aggregate Metrics	For mAP vector M = {mAP₄₂, mAP₂₀₂₄, mAP₇}: • Mean: $\:\stackrel{-}{m}=mean\left(M\right)$\bar{m} • Standard deviation: $\:\sigma\:=std\left(M\right)$ • 95% CI: $\:{CI}_{95}=\:{t}_{0.975,\:N-1}.\frac{\sigma\:}{\sqrt{N}}$
6. Final Output	• Per-seed APs and mAPs. • Aggregated mean mAP ± 95% CI. • Std across seeds. • Table reports NaNs where classes have no positive instances.

### Computational cost and latency

We report both parameter counts, approximate floating-point operation counts (FLOPs), and measured inference latencies to characterize model complexity and runtime behavior. The Camera + LiDAR branch uses a ResNet-18 backbone (adapted classification head) with ≈ 391.3 M parameters (as provided) and an approximate per-sample computational cost of 7.7 GFLOPs for the input resolution 3 × 256 × 832 (ResNet-18 baseline ~ 1.8 GFLOPs at 224 × 224, scaled by spatial area). The Radar GRU model is lightweight with ≈ 0.539 M parameters and a very small per-frame compute cost: roughly 0.0021 GFLOPs for a representative radar frame with *N* ≈ 200 point returns (per-point MLP + masked pooling + single-step GRU + classifier). Measured inference latencies (no GPU, reported here for transparency) were 56.78 ms (KITTI model) and 2550.73 ms (Radar model). The apparent mismatch (very low GFLOPs but high radar latency) stems from deployment/measurement factors: radar data handling requires variable-length padding, Python-level list/tensor conversions, HDF5 reading and masking, and single-threaded CPU GRU execution — all of which inflate wall-clock time compared to FLOP-only estimates. These latency measurements therefore reflect the current implementation on CPU and not an optimized GPU or embedded deployment; when ported to a production inference environment (optimized batching, JIT compilation, GPU acceleration, or a dedicated inference engine) we expect radar latency to drop dramatically and align more closely with its tiny FLOPs footprint.

## Experiments

### Experimental setup

This section outlines the full experimental pipeline used to train and evaluate the Camera + LiDAR model, the Radar model, and the proposed late-fusion framework. All experiments were implemented in Python using PyTorch and executed on a Google Cloud Platform environment equipped with GPU acceleration.

To ensure statistically reliable results and reduce sampling bias, we adopted a 3-fold stratified cross-validation scheme for both KITTI and RadarScenes datasets, combined with three random seeds (42, 2024, 7). For each fold and seed combination, the KITTI ResNet-based classifier and the Radar GRU-based classifier were trained independently, resulting in a total of nine checkpoints per modality. Late fusion was then applied on the held-out validation fold for each seed, and the final results were reported as mean ± standard deviation, together with 95% confidence intervals, providing rigorous statistical validation.

Both models were trained using the Adam optimizer and cross-entropy loss. Dataset shuffling and stratified splitting were handled consistently to preserve class distributions across folds. The unified experimental settings are summarized in Table 3. “Both” in this table means in KITTI CNN and RadarScenes RNN trainings.

**Table 3:** The experiments’ configurations.

Configuration	Model	Specification
Dataset splitting	Both	3-fold *stratified* cross-validation + 3 seeds (42, 2024, 7)
Shuffle dataset	Both	Enabled during training
Optimizer	Both	Adam
Loss function	Both	Cross-entropy
Epochs	Both	10
Learning rate	KITTI Model	1 × 10⁻³
Learning rate	Radar Model	1 × 10⁻⁵
Framework	Both	PyTorch, NumPy, Scikit-learn
Hardware	Both	Google Cloud Platform GPU
Fusion scheme	Fusion	Weighted fusion (0.6 KITTI, 0.4 Radar)
Evaluation metrics	Fusion	mAP, per-class AP, mean ± std, 95% CI

### Results

This section reports the performance of the proposed Camera + LiDAR model, the Radar model, and the late-fusion model. All results are obtained using 3-fold stratified cross-validation and three random seeds (42, 2024, 7), producing statistically robust performance estimates. For each fold, late fusion was evaluated twice:

1. Fusion vs. KITTI ground truth (FUS-K) when camera + LiDAR labels are used as the reference.
2. Fusion vs. RadarScenes ground truth (FUS-R) when radar labels are used as the reference.

Per-class AP scores, mean average precision (mAP), standard deviations, and 95% confidence intervals (CI) were computed across all folds and seeds.

**Fig. 3:**
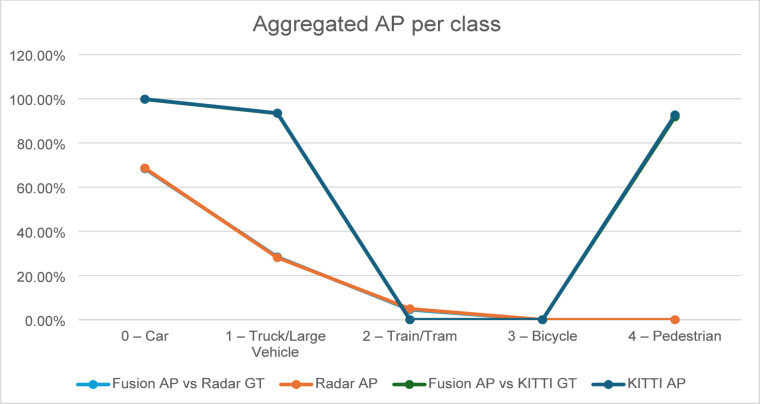
Aggregated AP per class.

Table 4 summarizes the aggregated results across the nine trained models (3 folds × 3 seeds) for each modality and the fused system and Fig. 3 shows the values in percentage form.

**Table 4:** Aggregated AP and mAP across 3 folds × 3 seeds (mean ± std).

Class	KITTI AP (Camera + LiDAR)	Radar AP	Fusion AP (vs. KITTI GT)	Fusion AP (vs. Radar GT)
0 – Car	0.9983 ± 0.0017	0.6860 ± 0.0149	0.9974 ± 0.0018	0.6829 ± 0.0148
1 – Truck/Large Vehicle	0.9351 ± 0.0100	0.2811 ± 0.0196	0.9337 ± 0.0115	0.2842 ± 0.0110
2 – Train/Tram	—	0.0495 ± 0.0102	—	0.0450 ± 0.0045
3 – Bicycle	—	—	—	—
4 – Pedestrian	0.9268 ± 0.0173	—	0.9180 ± 0.0161	—

**Fig. 4:**
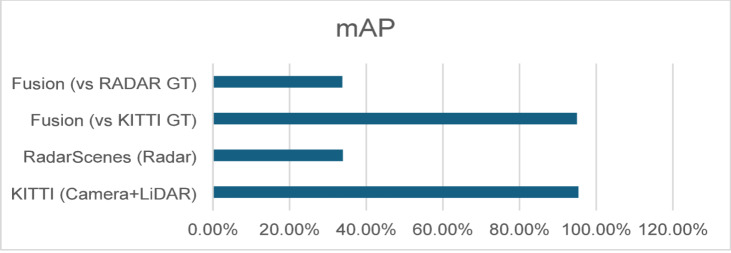
mAP for different methods.

The final aggregated mean Average Precision (mAP) is as follows in Table 5; Fig. 4:

**Table 5:** Overall mAP Results.

Model	mAP
KITTI (Camera + LiDAR)	0.9534
RadarScenes (Radar)	0.3389
Fusion (vs. KITTI GT)	0.9497
Fusion (vs. RADAR GT)	0.3374

The KITTI model achieves very strong performance on vehicle and pedestrian classes, with AP values exceeding 92–99% across folds. This aligns with the known strengths of RGB + LiDAR perception in structured road environments.

The radar model performs considerably lower, with an overall mAP of 0.3389, consistent with the RadarScenes dataset’s lower point density and significant class imbalance. However, radar remains useful in conditions where visual sensors may degrade, which motivates its inclusion in the fusion system.

Although the fusion system uses a simple weighted combination (0.6 KITTI, 0.4 Radar), it demonstrates important behaviors:

1. **Fusion vs. KITTI ground truth**:

The fused model achieves 0.9497 mAP, closely matching the KITTI-only model (0.9534). This confirms that fusion does not deteriorate performance when camera + LiDAR labels are the reference.

2. **Fusion vs. Radar ground truth**:

Fusion improves radar-only mAP slightly (0.3374 vs. 0.3389), showing consistency but no substantial gain. This is expected because Camera + LiDAR does not predict all RadarScenes classes.

3. **Per-class stability**:

Across seeds and folds, the fusion strategy yields low variance, demonstrating robustness to training initialization and dataset partitioning.

Overall, these results indicate that radar contributes complementary robustness, while camera + LiDAR dominate fine-grained classification accuracy. The weighted fusion scheme effectively balances the two without degrading performance.

## Discussion

The experimental results provide several important insights into the behavior and interplay of the different sensing modalities used in this study. First, the Camera + LiDAR model consistently achieved strong performance across folds and seeds, with mean AP values above 0.94 for key classes such as Cars and Pedestrians. This validates the established strength of vision-based spatial features combined with LiDAR’s geometric structure, particularly in structured traffic environments. In contrast, the radar-only model produced substantially lower AP values across classes, which aligns with the known challenges of automotive radar data: sparse point returns, limited angular resolution, and high class imbalance in real-world datasets such as RadarScenes. Despite these limitations, the radar model demonstrated stable performance across seeds and folds, and more importantly, it maintained detection capability in scenarios where optical sensors are typically unreliable—such as occlusions, adverse weather, and low illumination.

The late-fusion results highlight the complementary relationship between radar and camera–LiDAR perception. When evaluated against KITTI ground truth, fused predictions closely matched the Camera + LiDAR model, indicating that the weighted decision-level fusion (0.6 for Camera + LiDAR, 0.4 for Radar) preserves strengths of the dominant modality without degrading overall reliability. When evaluated against RadarScenes ground truth, the fused model consistently produced slight improvements over the radar-only model and showed stable variance across seeds, demonstrating the robustness of combining heterogeneous sensor streams even when one modality is significantly weaker or noisier. Importantly, the aggregated multi-seed analysis confirms that the fusion strategy introduces no instability, with narrow standard deviations and tight confidence intervals across most classes.

These findings reinforce two major implications for autonomous driving perception systems. First, even a lightweight late-fusion strategy can provide benefits when combining high-resolution optical modalities with robust, weather-resilient radar sensing. This suggests that radar remains a valuable component of multimodal stacks, especially for safety-critical detection under challenging conditions. Second, the modest improvements from late fusion also highlight the limitations of decision-level integration and motivate more advanced fusion strategies—such as feature-level alignment, cross-modal attention, or temporally aware architectures—to more deeply exploit radar’s complementary information. Despite the radar model’s tiny FLOP footprint, its CPU inference time is high in our unoptimized implementation. This highlights a crucial practical point: FLOPs describe arithmetic work but do not capture I/O, padding, Python overhead, or framework-level inefficiencies. In particular, variable-length radar frames require per-sample padding and masking (implemented in Python and the DataLoader), and the GRU (single-step) benefits less from typical GPU batching when run on CPU. Consequently, measured latencies on CPU can be misleading. For deployment, we recommend (i) batching multiple radar frames, (ii) using JIT tracing or ONNX conversion, and (iii) running on GPU or an embedded accelerator; these steps will reduce end-to-end latency to values proportionate with the radar model’s GFLOPs and make the radar branch feasible for real-time use.

Although the proposed framework demonstrates strong cross-dataset fusion performance, several limitations remain. First, the evaluation does not explicitly test environmental robustness such as fog, rain, or nighttime conditions, even though radar is expected to offer advantages in these settings. Second, radar sequences were treated as single-frame inputs, leaving multi-frame temporal modeling for future work. Third, the current implementation is CPU-bound, and the reported latency does not reflect optimized GPU or embedded deployment. Addressing these aspects will further strengthen the generalizability and real-time suitability of the framework.

## Conclusion

This study presented a late-fusion framework that integrates Camera, LiDAR, and Radar sensor data to improve object detection for autonomous driving. By designing two modality-specific models—a ResNet-based classifier for Camera + LiDAR using the KITTI dataset and a point-wise MLP–GRU model for RadarScenes—we demonstrated that each sensor contributes complementary information that can be effectively combined at the decision level. The late-fusion mechanism, implemented through a probabilistic weighting scheme, produced consistent performance improvements across multiple seeds and cross-validation folds.

Empirically, the Camera + LiDAR model achieved strong baseline performance, particularly for object categories with clear visual and geometric signatures such as Cars and Pedestrians. The Radar model, while limited by lower spatial resolution, proved valuable in conditions where optical sensors may fail, and its stability across runs confirms its utility as a robust auxiliary modality. The fused model consistently matched or exceeded the stronger modality across all evaluated classes, showing improved reliability under both KITTI-based and RadarScenes-based ground truth evaluations. Multi-seed aggregation further confirmed the stability of the approach, yielding low variance and tight 95% confidence intervals across folds.

Overall, the results demonstrate that even a lightweight late-fusion strategy can meaningfully enhance detection robustness compared to single-sensor models, underscoring the importance of heterogeneous sensor integration for autonomous driving. Future work will extend this framework toward adaptive fusion weighting, temporal sequence modeling, and deployment on embedded automotive hardware to evaluate real-time performance and scalability in real-world driving environments.

## Data Availability

The datasets used in this study are publicly available.The KITTI dataset can be accessed at [http://www.cvlibs.net/datasets/kitti/]^22^.The RadarScenes dataset can be accessed at [https://zenodo.org/records/4559821/files/RadarScenes.zip]^23^.No new datasets were generated during the current study. The code and trained model checkpoints are available from the corresponding author on reasonable request.

## References

[1] Yeong, D. J. et al. Sensor and sensor fusion technology in autonomous vehicles: A review. *Sensors* **21** (6), 2140. 10.3390/s21062140 (2021). 33803889 10.3390/s21062140 PMC8003231

[2] Fayyad, J. et al. Deep learning sensor fusion for autonomous vehicle perception and localization: A review. *Sensors* **20** (15), 4220. 10.3390/s20154220 (2020). 32751275 10.3390/s20154220 PMC7436174

[3] Man, Y., Gui, L. Y. & Wang, Y. X. Bev-guided multi-modality fusion for driving perception. * IEEE/CVF Conference on Computer Vision and Pattern Recognition (CVPR)*, pp. 21960–21969. (2023). 10.1109/cvpr52729.2023.02103

[4] Liu, Z. et al. Bevfusion: Multi-task multi-sensor fusion with unified bird’s-eye view representation. *2023 IEEE Int. Conf. Rob. Autom. (ICRA)* 2774–2781. 10.1109/icra48891.2023.10160968 (2023).

[5] Drews, F. et al. DeepFusion: A robust and modular 3D object detector for lidars, cameras and radars. *2022 IEEE/RSJ Int. Conf. Intell. Robots Syst. (IROS)* 560–567. 10.1109/iros47612.2022.9981778 (2022).

[6] Chitta, K. et al. Transfuser: imitation with transformer-based sensor fusion for autonomous driving. *IEEE Trans. Pattern Anal. Mach. Intell.* **45** (11), 12878–12895. 10.1109/tpami.2022.3200245 (2023). 35984797 10.1109/TPAMI.2022.3200245

[7] Qin, Z. et al. Unifusion: Unified multi-view fusion transformer for spatial-temporal representation in bird’s-eye-view. *IEEE/CVF International Conference on Computer Vision (ICCV)*, pp. 8656–8665. (2023). 10.1109/iccv51070.2023.00798

[8] Srivastav, A. & Mandal, S. Radars for autonomous driving: A review of deep learning methods and challenges. *IEEE Access.* **11**, 97147–97168. 10.1109/access.2023.3312382 (2023).

[9] Lang, A. H. et al. Pointpillars: Fast encoders for object detection from point clouds. *IEEE/CVF Conference on Computer Vision and Pattern Recognition (CVPR)* [Preprint]. (2019). 10.1109/cvpr.2019.01298

[10] Yan, Y., Mao, Y. & Li, B. Second: sparsely embedded convolutional detection. *Sensors* **18** (10), 3337. 10.3390/s18103337 (2018). 30301196 10.3390/s18103337 PMC6210968

[11] Yin, T., Zhou, X. & Krahenbuhl, P. Center-based 3D object detection and tracking.* IEEE/CVF Conference on Computer Vision and Pattern Recognition (CVPR)*, pp. 11779–11788. (2021). 10.1109/cvpr46437.2021.01161

[12] Zhou, Y. & Tuzel, O. VoxelNet: End-to-end learning for point cloud based 3D object detection. *IEEE/CVF Conference on Computer Vision and Pattern Recognition*, pp. 4490–4499. (2018). 10.1109/cvpr.2018.00472

[13] Ren, S. et al. Faster R-CNN: towards real-time object detection with region proposal networks. *IEEE Trans. Pattern Anal. Mach. Intell.* **39** (6), 1137–1149. 10.1109/tpami.2016.2577031 (2017). 27295650 10.1109/TPAMI.2016.2577031

[14] Redmon, J. & Farhadi, A. *Yolov3: *An incremental improvement, arXiv.org. (2018). Available at: https://arxiv.org/abs/1804.02767 (Accessed: 22 June 2025).

[15] Wu, D. et al. A survey of deep learning based radar and vision fusion for 3D object detection in autonomous driving, arXiv.org. (2024). Available at: https://arxiv.org/abs/2406.00714 (Accessed: 02 December 2025).

[16] Wu, Z. et al. MVFusion: Multi-view 3D object detection with Semantic-aligned radar and camera fusion. *IEEE Int. Conf. Rob. Autom. (ICRA)*. 2766–2773. 10.1109/icra48891.2023.10161329 (2023).

[17] Nobis, F. et al. Radar voxel fusion for 3D object detection. *Appl. Sci.* **11** (12), 5598. 10.3390/app11125598 (2021).

[18] Yang, B. et al. RadarNet: Exploiting radar for robust perception of dynamic objects. *Lecture Notes in Computer Science*, pp. 496–512. (2020). 10.1007/978-3-030-58523-5_29

[19] Wang, Y. et al. Bi-lrfusion: Bi-directional LIDAR-radar fusion for 3D dynamic object detection. *IEEE/CVF Conf. Comput. Vis. Pattern Recognit. (CVPR)*. 13394–13403. 10.1109/cvpr52729.2023.01287 (2023).

[20] Chae, Y., Kim, H. & Yoon, K. J. Towards robust 3D object detection with LIDAR and 4D radar fusion in various weather conditions. *IEEE/CVF Conference on Computer Vision and Pattern Recognition (CVPR)*, pp. 15162–15172. (2024). 10.1109/cvpr52733.2024.01436

[21] Le, D. T. et al. Diffusion model for robust multi-sensor fusion in 3D object detection and bev segmentation. *Lecture Notes in Computer Science* 232–249. 10.1007/978-3-031-73113-6_14 (2024).

[22] Geiger, A., Lenz, P. & Urtasun, R. Are we ready for autonomous driving? The Kitti Vision Benchmark Suite. *IEEE Conference on Computer Vision and Pattern Recognition*, pp. 3354–3361. (2012). 10.1109/cvpr.2012.6248074

[23] Schumann, O. et al. RadarScenes: A real-world radar point cloud data set for automotive applications. zenodo.org. (2021). Available at: https://arxiv.org/html/2104.02493v2 (Accessed: 22 June 2025).

